# Social media influencers in the space of pregnancy and parenting: a scoping review protocol

**DOI:** 10.1136/bmjopen-2024-087200

**Published:** 2024-10-18

**Authors:** Lucy Hives, Emma P Bray, Rebecca Nowland, Gill Thomson

**Affiliations:** 1 Maternal, Parental and Infant Nutrition and Nurture Research Team, School of Nursing and Midwifery, University of Central Lancashire, Preston, UK; 2 Stroke Research Team, School of Nursing and Midwifery, University of Central Lancashire, Preston, UK

**Keywords:** Social Media, Pregnancy, Public health, Parents

## Abstract

**Abstract:**

**Introduction:**

Social media influencers (SMIs) are popular sources of online information on various topics, including many aspects of health. Recently, there has been an upsurge in SMIs creating content about pregnancy and parenting, including from midwives, pregnant women and parents. Despite its popularity, SMI content on pregnancy and parenting is not currently regulated, which allows for misinformation and potential harm to women and their children. Research has also found that most women do not discuss the information they access online with their healthcare providers.

This is the first scoping review to map the existing evidence on SMIs in the context of pregnancy and early parenting.

**Methods and analysis:**

The scoping review will be conducted from May to December 2024 and reported using guidance from Arksey and O’Malley and the Preferred Reporting Items for Systematic Reviews and Meta-Analyses extension for Scoping Reviews checklist. 10 academic databases will be searched for relevant studies, using keywords and subject headings for the concepts of “social media”, “influencers”, “pregnancy” and “parenting.” All primary and secondary research studies of pregnancy and early parenting SMIs will be included. Two authors will screen the identified studies for eligibility. The risk of bias of the included studies will not be assessed. Extracted data will be presented in tables and will be described narratively.

**Ethics and dissemination:**

Ethical approval was not needed for this scoping review. Results will be published in a peer-reviewed journal, presented at conferences, posted on social media and presented to relevant groups.

**Registration details:**

The review is registered with the Open Science Framework (https://osf.io/7v4qbhttps://osf.io/7v4qb)

**STRENGTHS AND LIMITATIONS OF THIS STUDY**

- Members of the public have contributed to the design of this scoping review, including discussing the importance of this research area and suggesting database search terms.
- A vast range of databases will be searched to identify the existing primary and secondary research.
- Studies not published in English will be translated using freely available software (Google Translate) as possible.
- As this is a scoping review, quality assessment of the included studies will not be undertaken.

## Introduction

### Pregnant women and Internet use

 The information parents receive and the decisions they make during the critical first 1000 days (from conception to age 2) can have a lifelong impact on their child’s health.^1^ Many pregnant women, especially those who are younger and those expecting their first child,^2^ use the Internet to find resources about pregnancy and parenting.^3^ The information women access online includes foetal development at different gestational stages, nutrition and physical activity during pregnancy, and giving birth and infant feeding.^4^ Internet use for this purpose is increasing,^4^ with the majority (84–97%) of pregnant women reporting ever having searched for such information online.^5^ Women search the Internet frequently and perceive this information to be useful, important and reliable.^5 6^

The reasons women give for accessing pregnancy and parenting information online include ease and speed of access, wanting to find people in similar situations, sharing experiences, seeking reassurance, feeling ashamed or embarrassed to speak to a healthcare professional, long waiting times for appointments, short appointments and lack of formal information resources.^46^,^8^ Social media, such as Facebook groups and blogs, can be helpful sources of information, advice and peer support and can help to increase parental self-confidence and reduce social isolation.^9 10^

Despite the perceived benefits of Internet resources, women can experience information overload and difficulty navigating, often conflicting, information.^11^ There is also a wealth of incorrect and non-evidence-based information online,^12^ making it difficult to know what information to trust, posing risks to both the mother and child. This risk may be worsened by women’s reluctance to speak to maternity care professionals about the information they access online.^5^

### Social media influencers (SMIs)

A popular source of online health information is SMIs.^13^,^17^ SMIs range from nano-influencers (with 1000 to 9999 followers) to mega-influencers (with over 1 million followers),^18^ each with the potential to impact the attitudes, behaviours and decision-making of their followers who may be members of their local communities or wider populations. Many SMIs are paid or receive incentives to promote brands and products to their followers. A recent survey revealed that 49% of Internet users rely on SMI recommendations and 40% have bought a product after seeing it advertised on Instagram, Twitter or YouTube.^19^

Despite recently introduced rules requiring SMIs to disclose when they are being paid or receiving incentives to advertise products,^20^ health content from SMIs remains unregulated. The COVID-19 pandemic also drew attention to the spread of misinformation on social media and its negative consequences. For example, some SMIs have undermined public health information, resulting in an unwillingness to accept treatments, for example, COVID-19 vaccines.^21^

### Pregnancy and parenting SMIs

Recently, there has been an upsurge of SMIs creating pregnancy- and parenting-related content. Videos and other media posted by SMIs can have viewer counts in the millions,^22^ and one study reported that 89% of new mothers used social media sites to ask questions and receive advice relating to pregnancy and parenting.^23^ Women not yet considering trying to conceive may also be exposed to this information due to the amount of content available and the algorithm suggesting content for them to view.^22^

Some SMIs in this space are qualified health professionals^24^ (eg, midwives, health visitors, lactation consultants and sleep experts), and others are members of the public who are pregnant or parents themselves.^25^ As well as mothers, there are also a small number of fathers creating online content.^26^ Information shared by SMIs may or may not be evidence-based or may be based on their personal experiences, which may include idealised and unrealistic views of pregnancy and parenting.^27^

A vast range of pregnancy and parenting content is available from SMIs. Women in their first and third trimesters and women expecting their first baby may have greater information needs and may, therefore, use the Internet for this purpose more often.^28^,^30^ SMIs also speak about birth control,^31^ difficulty conceiving and IVF,^32^ and miscarriage,^33^ all of which are topics within the scope of this review.

There are no existing scoping reviews that explore the breadth of research on pregnancy and parenting content from SMIs; however, a recent systematic review^34^ of 17 studies (six of which were specific to SMIs) investigated how SMIs and bloggers might impact experiences of and decision-making during pregnancy and parenthood. Multiple benefits (eg, increased happiness, support and parental self-efficacy) and harms (eg, fear of missing out, envy towards the SMI and social comparison) of SMI content on pregnant women and parents were found. Certain groups may be particularly reliant on and vulnerable to the impacts of social media, for example, pregnant adolescents and adolescent parents,^35^ and migrant and ethnic minority populations.^36^

It is important that healthcare professionals are aware of the information women are accessing from SMIs regarding pregnancy and parenting. This is so that they can have open and supportive conversations with parents and moderate the information they are receiving to ensure that they are following the best available evidence and not compromising their or their baby’s health. This is especially the case as there is evidence that women feel unable to speak to their midwives and health visitors about what they are accessing online.^5^

This scoping review will examine the extent, range and nature of the available research in the area of pregnancy and parenting SMIs. Additionally, it will determine the value of undertaking further systematic reviews and highlight any gaps in the literature where further primary research is needed. Building on the existing systematic review conducted in this area,^34^ this scoping review will include a much broader range of SMI content (eg, conception and pregnancy loss) and outcomes (beyond pregnant women and parents’ experiences and the impact of SMIs). This scoping review will provide a comprehensive understanding of the range of SMI content being created, who creates this content, how SMI content is being used, why it is being used and by which populations.

### Review question

What is the existing evidence on SMIs in relation to pregnancy and early parenting?

## Methods and analysis

### Study design

The scoping review has been registered on the Open Science Framework (https://osf.io/7v4qb). It will be conducted and reported using guidance from Arksey and O’Malley^37^ and in line with the Preferred Reporting Items for Systematic Reviews and Meta-Analyses extension for Scoping Reviews^38^ checklist and guidance. Any changes to the protocol will be detailed in the final scoping review article. The scoping review will be conducted from May to December 2024.

### Search strategy

The search strategy for the scoping review was adapted from a similar search,^39^ which was codesigned by the research team, an information specialist, SMIs and members of the public who access content from SMIs. The search includes subject headings and keywords for the concepts of “social media”, “influencers”, “pregnancy” and “parenting”.

A preliminary search of the academic database Medline was undertaken to identify relevant articles. Additional text words and index terms identified from the title and abstract of relevant articles, and suggested by the research team, were incorporated into the final search strategy for Medline (online supplemental appendix 1). The search strategy will be adapted for use in each database and registry as appropriate.

Comprehensive searches of the following 10 academic databases will be conducted: Medline and Epub Ahead of Print, In-Process, In-Data-Review and Other Non-Indexed Citations and Daily (Ovid), Embase (Ovid), Cumulated Index in Nursing and Allied Health Literature (CINAHL) Ultimate (EBSCOhost), American Psychological Association (APA) PsycINFO (EBSCOhost), Academic Search Complete (EBSCOhost), Computers and Applied Sciences Complete (EBSCOhost), WHO Global Index Medicus, the Cochrane Library, Scopus (Elsevier) and ProQuest Dissertations and Theses. As well as academic databases, the following registries will be searched to identify any registered ongoing primary and secondary research studies: International Standard Randomised Controlled Trial Number (ISRCTN) Registry, Clinicaltrials.gov, International Clinical Trials Registry Platform Search Portal, PROSPERO and Open Science Framework. The reference lists and citations of all included studies will be screened and Research Rabbit (https://www.researchrabbit.ai/) will be used to identify any relevant articles that may have been missed.

### Study selection

Studies identified during the database searches will be imported into EndNote (V.X9, Clarivate Analytics, Philadelphia, PA), where they will be deduplicated. The screening process will be conducted using Rayyan (https://www.rayyan.ai/). Two independent reviewers will screen all titles and abstracts, and then all remaining full-text articles. Disagreements will be resolved through discussion, where necessary with the help of a third reviewer. The study selection process will be documented in a PRISMA flow diagram.

### Eligibility criteria

#### Participants/population

Studies involving members of the public who access pregnancy and parenting content from SMIs will be included. Studies of SMIs (either described as such in the article or with at least 1000 followers on a social media platform) who create content relating to pregnancy and parenting, and who may be qualified health professionals or members of the public, will also be included. Studies of participants of any age and gender will be included. Studies of all levels of SMI will be included (ranging from nano- to mega-influencers). Studies about social media more broadly, rather than specifically on SMIs, will be excluded.

#### Intervention(s), exposure(s)

Studies of all aspects of pregnancy and parenting content from SMIs will be included for example, trying to conceive, stages of pregnancy, miscarriage, birth, birth trauma, baby loss and infant feeding. SMI content on parenting will only be included if it targets parents with children up to the age of 2. Studies of SMIs content not relating to pregnancy or parenting will be excluded. Studies of pregnancy and parenting content not created and shared by SMIs will not be included.

#### Comparator(s)/control

Studies with or without a comparator will be included.

#### Outcome(s)

Any outcomes will be included.

### Study design

Any published primary and secondary research studies will be included. As this is a relatively new area of research, dissertations and theses will be included. Conference abstracts, protocol papers and study registrations will be listed if full-text articles are not available. Editorials, commentaries, erratum, expert opinion papers, non-systematic literature reviews and book chapters will be excluded.

### Setting

Study setting will be online social media platforms, including but not limited to Instagram, TikTok and YouTube. Studies of blogs and blogging websites will be excluded. Studies from all countries will be included.

### Years

All years of publication will be considered, although from the preliminary database search and the lead authors’ knowledge of this area of research, it is unlikely that there are any articles in this area published prior to 2019.

### Language

Studies published in all languages will be included where possible. Those not published in English will be translated using freely available software (Google Translate).

### Data extraction (selection and coding)

One researcher will extract data from the included studies, and this will be checked by another researcher. The following data will be extracted.

1. Article information: first author, year of publication, study title, journal and country of publication.
2. Design and methods: study aim, study design, study methods (quantitative, qualitative and mixed methods) and data collection methods (eg, questionnaires, interviews and focus groups).
3. Exposure: SMI demographics (healthcare professional/member of the public, number of followers, age, gender, ethnicity and country), topic of social media content (eg, exercise before and after birth, infant feeding and sleep), social media platform and content media (eg, video, photograph).
4. Participants: number of participants, participant characteristics (influencer or follower) and follower demographics (age, gender, ethnicity, country and pregnancy/parent status).
5. Any findings.

### Risk of bias (quality) assessment

In line with scoping review methodology,^37 38^ a quality assessment of the included studies will not be conducted. Scoping reviews are concerned with mapping the existing research in a particular area, irrespective of the quality of included studies.^38^

### Strategy for data synthesis

It is anticipated that quantitative, qualitative and mixed methods studies will be included in the scoping review. Data extracted from included studies will be collated and tabulated. Content analysis^40^ will be used to identify the different SMI content topics and types of SMIs (eg, healthcare professional/parent and nano-/micro-/macro-/mega-influencer) within the included literature. A convergent synthesis design^41^ will be used to analyse the findings. This will involve (a) the quantitative findings being converted into narrative summaries; (b) the quantitative summaries being combined with the qualitative findings; and (c) the findings being organised into themes that represent the whole dataset.^41^ Team members have expertise in using a convergent-type approach when analysing data from scoping reviews.^40 42^

### Patient and public involvement

Members of the public have been involved throughout this programme of research, funded by UCLan’s Research Institute for Global Health and Well-Being and the Research Design Service North–West. Many discussions have taken place with members of the public who access content from SMIs, around their experiences of accessing this content, any issues they have faced and the importance of this research area. Conversations have also taken place with SMIs who create pregnancy and parenting content. The public and SMIs have contributed to the search strategy for the review.

## Ethics and dissemination

As this is a scoping review, ethical approval is not required. The review is registered on the Open Science Framework (https://osf.io/7v4qb). The completed review will be published in a peer-reviewed journal, presented at conferences and to members of the public, and will be publicised on social media.

## Conclusion

This scoping review will be the first to map the existing evidence on SMIs in the context of pregnancy and early parenting in the first 1000 days. It will identify any gaps where future research is needed, as well as areas where systematic reviews are warranted. By synthesising the existing research in this area, we can begin to understand why and how people are using this kind of online content. This work will highlight the types of information and support that parents seek, and which may not currently be provided via universal healthcare.

## supplementary material

10.1136/bmjopen-2024-087200 online supplemental file 1
