# Supplementary material for: Social media influencers in the space of pregnancy and parenting: a scoping review protocol
Source: BMJ Open. 2024 Oct 18;14(10):e087200. doi: 10.1136/bmjopen-2024-087200 (PMC11492936; doi:10.1136/bmjopen-2024-087200)
Supplement: online supplemental file 1 [file bmjopen-14-10-s001.docx]

**Ovid MEDLINE(R) and Epub Ahead of Print, In-Process, In-Data-Review & Other Non-Indexed Citations and Daily 1946 to April 3, 2024**

Search run on 3^rd^ April 2024

| **#** | **Search** | **Results** |
| --- | --- | --- |
| 1 | Exp Pregnancy/ | 1027639 |
| 2 | Parenting/ | 21976 |
| 3 | Exp Parents/ | 147830 |
| 4 | Pregnan* OR Birth* OR Postnatal* OR Perinatal* OR Prenatal* OR Parent* OR Mother* OR Father* OR Mum OR Mummy OR Dad OR Daddy OR Family OR Families OR Trimester* OR Antenatal OR Ante-natal OR Prenatal OR Pre-natal OR Postpartum OR Post-partum OR Postnatal OR Post-natal OR Intrapartum OR Intra-partum OR Perinatal OR Peri-natal OR Childbirth* OR Maternal | 3535760 |
| 5 | Influencer* OR Vlogger OR Microcelebrit* OR Micro celeb* OR Megainfluencer* OR Macroinfluencer* OR Microinfuencer* OR Nanoinfluencer* OR YouTuber OR You Tuber OR Instagrammer OR Instaauthor OR Instafamous OR Instawriter OR Opinion leader* OR Content creat* OR Brand ambassador* | 4493 |
| 6 | Mumfluencer* OR Momfluencer* OR Sharent* OR Instaparent* | 16 |
| 7 | Social Media/ | 17105 |
| 8 | Online Social Networking/ | 320 |
| 9 | Internet/ | 82528 |
| 10 | Social media OR Social network* OR Social technolog* OR Online OR On line OR Internet OR Web OR Website OR Digital OR Cyberspace OR Cyber space OR Webcast* OR Podcast* OR Vlog OR Vlogging OR Bebo OR Douyin OR Facebook OR Flikr OR Instagram OR Kuaishou OR Likee OR LinkedIn OR Myspace OR PicsArt OR Pinterest OR QQ OR Quora OR Qzone OR Reddit OR Weibo OR Snapchat OR Tieba OR TikTok OR Tik Tok OR Tumblr OR Twitch OR Twitter OR WeChat OR YouTube OR You Tube OR OnlyFans OR HouseParty OR Mumsnet | 799712 |
| 11 | #1 OR #2 OR #3 OR #4 | 3543180 |
| 12 | #7 OR #8 OR #9 OR #10 | 799712 |
| 13 | #11 AND #5 | 828 |
| 14 | #13 OR #6 | 841 |
| 15 | #14 AND #12 | 194 |
