# Supplementary material for: Social media influencers in the space of pregnancy and parenting: a scoping review protocol
Source: BMJ Open. 2024 Oct 18;14(10):e087200. doi: 10.1136/bmjopen-2024-087200 (PMC11492936; doi:10.1136/bmjopen-2024-087200)
Supplement: Supplementary file 2 [file bmjopen-2024-087200.reviewer_comments.pdf]

PEER REVIEW HISTORY

BMJ Open publishes all reviews undertaken for accepted manuscripts. Reviewers are asked to complete a checklist review form and are provided with free text boxes to elaborate on their assessment. These free text comments are reproduced below.

ARTICLE DETAILS

Title (Provisional)

Social media influencers in the space of pregnancy and parenting: A scoping review protocol

Authors

Hives, Lucy; Bray, Emma P; Nowland, Rebecca; Thomson, Gillian

---

VERSION 1 - REVIEW

Reviewer 1

Name Chee, Rachelle

Affiliation Central Queensland University, School of Nursing, Midwifery & Social Sciences

Date 23-May-2024

COI I am the first author of the systematic review on a similar topic cited by the authors (33).

Thank you for the opportunity to review this scoping review protocol in an exciting area of future research. The protocol is clear and descriptive, providing sufficient purpose and rationale for the research. You have also appropriately described how this scoping review differs significantly from the pre-existing literature review you have cited. Your search strategy has been comprehensively described. I have only a few small suggestions and otherwise recommend this protocol for publication.

1. The cited literature review (33) included 17 studies, not 6 (see page 4, line 33).

2. Page 5, line 54 (under participants/population) the first two sentences of this paragraph sound as though participants are being selected for a primary research project, when you are in fact selecting studies in which there may have been participants. Revision for clarity is recommended.

3. Will you be considering a limitation based on the age of the children to whom the parenting content pertains, or perhaps a particular developmental stage? Covering pre-conception until adolescence will produce very heterogeneous results and is a significant task to screen and map.

4. There is a typo "Fwill" on line 59 of this page.

5. There is a typo on page 12 "microinfuencer" instead of "microinfluencer".

Congratulations on submitting a comprehensive protocol and I look forward to seeing its completion.

**Reviewer**	**2**
**Name**	**Hilty, Donald**
**Affiliation**	**Northern California Veterans Administration Health Care System, MH**
**Date**	**14-Jun-2024**
**COI**	**N/A**

General comments

1. Interesting and useful topic and study. Good plan with Arksey and Prisma. Scoping review indicated as previous systematic review not the best tool for identifying what is out there, where the holes in literature are and such.

2. Questions.

a. Need some measure on Risk of Bias, in general, and particularly with secondary studies?

i. Some groups are adapting syst rev methods: incomplete data, selective reporting, and such.

ii. What if the studies have qualitative methods but used 'poor' ones? This creates bias. Qualitative steps to analyze disparate populations, data and methods of studies can be used like: content, discourse and framework qualitative analysis techniques to analyze findings from papers and classify, summarize and tabulate the behavioral data; discourse and thematic analyses to search for themes and patterns; and framework analysis to sift through, chart and sort data in accordance with key issues and themes a series of steps (e.g., indexing; charting; mapping and interpretation). We adapted Crowe M, Inder M, Porter R. Conducting qualitative research in mental health: Thematic and content analyses. Aust N Z J Psychiatry. 2015;49(7):616-623. doi:10.1177/0004867415582053.

b. On this subject of qualitative methods, please clarify that there are mixed vs. quantitative methods? Need to say more than 'narrative description'. Qualitative methods are indicated and which ones are being used, and is a team member skilled? What steps or procedures will be used?

3. Presentation.

a. Readability: good, overall.

b. Flow/logical: good.

Specific comments

Title.

1. Good.

Abstract.

1. Time period?

2. Suggest adding some form of Risk of Bias assessment.

3. Helps to list inclusion criteria.

Introduction.

1. Could be more concise, yet good content. A basic approach is:

a. Relevance.

b. Current state.

c. The gap (that the manuscript will fill).

d. Objectives.

2.

Methods.

1. Good overall.

2. Agree with dissertations.

3. Methods/procedures. Data collection → table: include column or data on

a. Study limitations?

b. Their assessment of risk of bias.

Results.

1. N/A

Discussion.

1. N/A.

Conclusion.

1. TBA.

Tables/Figures

1. Plan okay; suggestions above

References

1. Okay.

VERSION 1 - AUTHOR RESPONSE

Dear Editor,

We thank yourself and the reviewers for their comments.

Please find attached a table of our responses.

Very best wishes,

Lucy

Editor/ reviewer comment	Response
(1) Please revise the 'Strengths and limitations of this study' section of your manuscript (after the abstract). This section should contain up to five short bullet points, no longer than one sentence each, that relate specifically to the methods. The novelty, aims, results or expected impact of the study should not be summarised here.	Thank you, we have made these changes.
(2) Please include the planned start and end dates for the study in the methods section.	Dates have now been added.
Thank you for the opportunity to review this scoping review protocol in an exciting area of future research. The protocol is clear and descriptive, providing sufficient purpose and rationale for the research. You have also appropriately described how this scoping review differs significantly from the pre-existing literature review you have cited. Your search strategy has been comprehensively described. I have only a few small suggestions and otherwise recommend this protocol for publication.	Thank you so much for your kind comments.
1. The cited literature review (33) included 17 studies, not 6 (see page 4, line 33).	Thank you for this comment. We have edited this sentence to make it clearer so that it now reads: "There are no existing scoping reviews that explore the breadth of research on pregnancy and parenting content from SMIs, however, a recent systematic review[33] of seventeen studies (six of which were specific to SMIs) investigated how SMIs and bloggers might impact experiences of and decision-making during pregnancy and parenthood."
2. Page 5, line 54 (under participants/population) the first two sentences of this paragraph sound as though participants are being selected for a primary research project, when you are in fact selecting studies in which there may have been participants. Revision for clarity is recommended.	Thank you, we have tweaked the wording of these two sentences for clarity.
3. Will you be considering a limitation based on the age of the children to whom the parenting content pertains, or perhaps a particular developmental stage? Covering pre-conception until adolescence will	Thank you very much for this helpful comment. We have added the following sentence to the inclusion

produce very heterogeneous results and is a significant task to screen and map.	criteria: "SMI content on parenting will only be included if it targets parents with children up to the age of two." We have also added to the introduction section, "The information parents receive and the decisions they make during the critical first 1,000 days (from conception to age two) can have a lifelong impact on their child's health".
4. There is a typo "Fwill" on line 59 of this page.	Thank you, we have corrected this typo.
5. There is a typo on page 12 "microinfuencer" instead of "microinfluencer".	Thank you, we have corrected this typo.
Congratulations on submitting a comprehensive protocol and I Iook forward to seeing its completion.	Thank you!
1. Interesting and useful topic and study. Good plan with Arksey and Prisma. Scoping review indicated as previous systematic review not the best tool for identifying what is out there, where the holes in literature are and such.	Thank you for your kind comments.
a. Need some measure on Risk of Bias, in general, and particularly with secondary studies?<br><br>i. Some groups are adapting syst rev methods: incomplete data, selective reporting, and such.<br>ii. What if the studies have qualitative methods but used 'poor' ones? This creates bias. Qualitative steps to analyze disparate populations, data and methods of studies can be used like: content, discourse and framework qualitative analysis techniques to analyze findings from papers and classify, summarize and tabulate the behavioral data; discourse and thematic analyses to search for themes and patterns; and framework analysis to sift through, chart and sort data in accordance with key issues and themes a series of steps (e.g., indexing; charting; mapping and interpretation). We adapted Crowe M, Inder M, Porter R. Conducting qualitative research in mental health: Thematic and content analyses. Aust N Z J Psychiatry. 2015;49(7):616-623. doi:10.1177/0004867415582053.	Thank you for your suggestion. We have stated in the "Risk of bias (quality) assessment" section that<br>"In line with scoping review methodology[36,37], a quality assessment of the included studies will not be conducted. Scoping reviews are concerned with mapping the existing research in a particular area, irrespective of the quality of included studies.[37]" We have added references from Arksey and O'Malley and PRISMA-ScR to justify this. This is also included in the limitations section of our paper.
b. On this subject of qualitative methods, please clarify that there are mixed vs. quantitative methods? Need to say more than 'narrative description'. Qualitative methods are indicated and which ones are being used, and is a team member skilled? What steps or procedures will be used?	Thank you for your comment. The review will include quantitative, qualitative and mixed methods studies as detailed in our methods section. As such, we have strengthened our strategy for data synthesis.
3. Presentation.<br>a. Readability: good, overall.<br>b. Flow/logical: good.	Thank you.
Specific comments<br>Title.<br>1. Good.	Thank you.

Abstract.<br>1. Time period?<br><br>2. Suggest adding some form of Risk of Bias assessment.<br><br>3. Helps to list inclusion criteria.	Thank you. We have added the time period of the review so that the abstract now reads: "The scoping review will be conducted from May to December 2024 and reported using guidance from Arksey and O'Malley and the PRISMA-ScR checklist."<br><br>Please see our response relating to risk of bias assessment above.<br><br>Unfortunately, there is no room in the word count to add more detail on the inclusion criteria. The abstract currently reads "All primary and secondary research studies of pregnancy and early parenting SMIs will be included."
Introduction.<br>1. Could be more concise, yet good content. A basic approach is:<br>a. Relevance.<br>b. Current state.<br>c. The gap (that the manuscript will fill).<br>d. Objectives.	Thank you. We have reviewed the introduction section which we feel meets all of your pointers. We have made this section more concise where we feel we were able to.
Methods.<br>1. Good overall.<br>2. Agree with dissertations.<br>3. Methods/procedures. Data collection → table: include column or data on<br>a. Study limitations?	Thank you, we have considered adding study limitations to the data extraction table, however we decided not to as scoping reviews are typically concerned with mapping the existing research that has been conducted, not on evaluating how well it has been done.
b. Their assessment of risk of bias.	Please see our response relating to risk of bias assessment above.
Results.<br>1. N/A	N/A
Discussion.<br>1. N/A.	N/A
Conclusion.<br>1. TBA.	We have now added a conclusion section.
Tables/Figures<br>1. Plan okay; suggestions above	Please see responses to these above.
References<br>1. Okay.	Thank you for checking.
